# A study of computed tomography scan and magnetic resonance imaging findings in pseudohypoparathyroidism

**DOI:** 10.4103/0972-2327.70870

**Published:** 2010

**Authors:** J. Kalita, N. Kumar, P. K. Maurya, U. K. Misra

**Affiliations:** Department of Neurology, Sanjay Gandhi Post Graduate Institute of Medical Sciences, Lucknow 226014, India

**Keywords:** CT scan, MRI, parathormone, pseudohypoparathyroidism

## Abstract

We report three children with pseudohypoparathyroidism aged 13-16 years who presented with seizures and tetany. CT scan revealed striatopallidal calcification in two. MRI revealed wide-spread involvement showing T1 hyperintensity in striatopallidodentate distribution in all three and midbrain in one patient. T2 and FLAIR images were normal. T1 hyperintensity could represent early stage of calcification in whom MRI is more sensitive.

## Introduction

Pseudohypoparathyroidism (PsHP) is a hereditary disorder characterized by signs and symptoms of hypoparathyroidism typically in association with distinct skeletal and developmental deficit. Hypoparathyroidism is due to deficient end organ response to parathormone (PTH). Hyperplasia of parathyroid, a response to hormone resistance, causes elevation of PTH level. Studies have shown variable clinical spectrum, pathophysiology, and genetic defects. On CT scan, striatopallidodentate calcification is typical.[1] Evaluation of calcification by MRI reveals either the knull effect or reduction of signal intensity.[2,3] There is paucity of MRI studies in PsHP. Some studies have reported increased T1 hyperintensity on MRI.[4,5] We report three patients with PsHP and highlight their CT and MRI findings.

## Case Reports

### Case 1

A 12-year-old boy, average student of class VII, presented with 1 year history of generalized tonic clinic seizures which lasted for 2-3 min. In the initial 2-3 days of his illness, he had 2-3 seizures daily for which he received carbamazepine 200 mg twice daily. Seizure though was controlled, but he started having carpopedal spasms lasting for 1-2 min. His elder brother aged 16 years also had similar illness. Examination revealed that the patient was of average built and nutrition (height 157.5 cm, weight 48kg) and there were no dysmorphic features. The neurological examination was normal except Trousseau’s sign.

The laboratory studies revealed normal hemoglobin and blood counts. His serum calcium was 6 (ionized 4) (normal 8.5-10.8, ionic 4.6-5.3) mg/dl, phosphorus 8 (normal 2.5-4.5) mg/dl, alkaline phosphatase 999 U/l, and magnesium 2.2 mg/dl, blood urea nitrogen 8 mg/dl, and creatinine 0.7 mg/dl. 24-h urinary calcium was 150 (normal 200) mg, phosphorous 0.06 (normal 1) g, and creatinine 1.29 (normal 1-2) g. His calculated TmP/GFR was 2.57 mmol/l (normal 1.15-2.44 mmol/l for 2-15 years age).[6] The glomerular filtration rate was 122 ml/min. Serum 25-OH vitamin D was 9.98 (normal 9-47) ng/ml and PTH 335.4 (normal 9-55) pg/ml. His electrocardiogram, electroencephalography, radiograph of hand, feet and pelvis were normal. CT scan revealed bilateral hyperdensity in bilateral caudate, globus pallidus, putamen, and right frontal white matter. Cranial MRI revealed T1 hyperintensity in caudate, globus pallidus, putamen, and dentate nuclei bilaterally. T2 and FLAIR images were normal. He was prescribed calcitrol 0.25 mg twice daily and calcium gluconate 500 mg four times daily without anticonvulsant, on which he was asymptomatic till 1 year follow up.

### Case 2

The brother of the first patient was aged 16 years. His height was 162.5 cm and weight 51 kg and also suffered from similar illness. He had normal pubertal characteristics and did not have any dysmorphic features. His investigations revealed serum calcium 5.56 mg/dl (ionized 4.9 mg/dl), phosphorous 7.6 mg/dl, alkaline phosphatase 271 U/L, serum protein 8.9 gm/dl (albumin 4.9 g/dl), serum creatinine 0.8 mg/dl, and blood urea nitrogen 8.9 mg/dl. 24-h urinary creatinine was 3.1 g, calcium 12 mg, and phosphorous 0.5 g. The calculated glomerular filtration rate (GFR) was 110 ml/min and TmP/GFR was 2.40 mmol/l (normal adult 0.8–1.35 mmol/l). His PTH level was 193.7 (normal 9-55) pg/ml, 25-OH vitamin D 26.22 (normal 9-47) ng/ml, follicle stimulating hormone 7.95 (normal 1.1-13.5) IU/l, luteinizing hormone 3.54 (normal 0.4-5.7) IU/l, thyrotropin 2.38 (normal 0.325) IU/l. His CT scan revealed bilateral calcification of globus pallidus, caudate, and putamen. Cranial MRI revealed increased T1 signal intensity in caudate, globus pallidus, and dentate nuclei bilaterally. T2 and FLAIR images were normal. He was treated with calcitrol and calcium carbonate.

### Case 3

**Figure 1:**
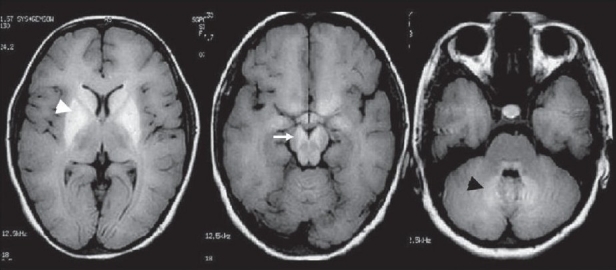
Cranial MRI on T1 sequence of the patient #3 with pseudohypoparathyroidism shows hyperintensity in the striatopallidal (white arrow head), midbrain (white arrow) and pontocerebellar region (black arrow head) whose CT scan was normal

A 13-year-old girl, average student of class VII, presented with history of tonic clonic seizures which lasted for 2 min, 10 days back. The seizures were preceded by colored fortification spectra lasting for 20-30 s. After cessation of seizures, she remained unconscious for 10 min. Her grandmother also suffered from tonic clonic seizures. She was of average built and nutrition (height= 147.5 cm, Wt=40 kg). She attained menarche at 12.6 years and there were no dysmorphic features. Her general and neurological examination was normal except for positive Trousseau’s sign. Her investigations revealed normal hemoglobin and blood counts. Serum calcium was 6.8 (corrected calcium 7.36) mg/dl, phosphorous 5.2 mg/dl, alkaline phosphatase 902 U/l, magnesium 1.9 mg/dl, albumin 3.3 gm/dl, creatinine 0.8 mg/dl, and BUN 12 gm/dl. 24-h urinary calcium was 100 mg, phosphorous 0.32 g, and creatinine 0.34 g. The calculated TmP/GFR was 1.439 mmol/l (normal 1.15-2.44 mmol/l for 2-15 years of age). The calculated glomerular filtration rate was 88.2 ml/min. Her PTH level was 348.3 pg/ml and 25-OH vitamin D was 5.06 ng/ml. Radiographs of hands, feet and pelvis were found to be normal. Cranial CT scan was normal but MRI revealed hyperintense signal changes on T1 in caudate, globus pallidus, putamen, midbrain, and dentate nucleus bilaterally. These lesions were isointense on T2 and FLAIR sequence [Figure 1]. She was prescribed calcitrol 0.25 mg daily and calcium carbonate 500 mg four times daily and was asymptomatic for 1 year.

**Figure 2:**
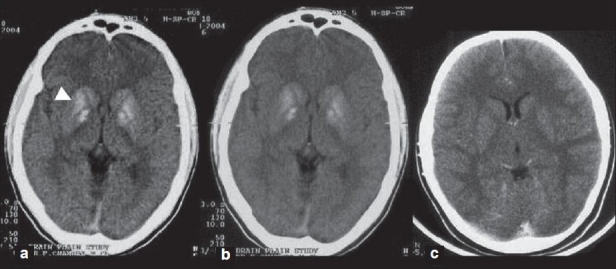
CT scans of the patients with pseudohypoparathyroidism showing striatopallidal calcifi cation in patient # 1 (a) and 2 (b) and the CT scan was normal in patient # 3 (c).

The radiological changes are summarized in Table 1 and shown in Figure 2.

**Table 1:** CT and MRI changes in patients with pseudohypoparathyroidism

	Patient 1	Patient 2	Patient 3
CT scan	Hyperdensity in RtF, BL C, GP, P	Hyperdensity in BL C, GP, P	Normal
MRI			
T1	Hyperintensity in BL C, GP, P, dentate	Hyperintensity in BL C, GP, P, dentate	Hyperintensity in BL C, GP, P, brain stem, dentate

Rt=right, BL=bilateral, C=caudate, GP=globus pallidus, P putamen.

## Discussion

The diagnosis of PsHP in our patients was based on clinical picture, hypocalcemia, hyperphosphatemia, raised serum PTH with normal renal functions, and magnesium level. Albright hereditary osteodystrophy is however unlikely as none of our patient had any dysmorphic features such as round face, short stature, obesity, brachycephaly, short and low set nasal bridge, and strabismus.. Urinary cAMP after a PTH challenge is the confirming test in PsHP and was not done in our patient.

Basal ganglia calcification has been described in various endocrinal (hypoparathyroidism, pseudohypoparathyroidsm, pseudopseudohypoparathyroidism), congenital or developmental (Fahr disease, Cockayne syndrome, tuberous sclerosis, oculocraniosomatic disease), inflammatory (cytomegalic inclusion disease, toxoplasma, cysticercosis), and toxic or hypoxic causes (CO poisoning, lead intoxication, therapeutic radiation, methotrexate therapy).[7] The clinical, biochemical, and other associated radiological findings are however different in different conditions. The typical intracranial striatopallidal calcification on CT scan was found in two of the three of our patients. However, MRI revealed signal changes on T1 in all three patients and revealed more extensive changes than CT scan. The diagnosis of calcification by MRI is regarded more difficult than CT scan when evaluated visually. On MRI, calcification results in reduction of signals which is due to reduced proton density.[4] CT documented calcification on MRI reveals a number of patterns: (1) no signal change on T1 and T2, (2) decreased signal intensity on T2 without any change on T1 image, and (3) increased signal intensity on T1 with spots of decreased signal changes on T2 sequence. These differences in signal intensities are supposed to reflect different conditions of calcium deposit.[5,8] Hyperintensity on T1 was found in all three of our patients, suggesting importance of this pattern. In patient #2, CT and MRI provided corresponding signal alteration in caudate, globus pallidus, and putamen, but in dentate nucleus MRI provided additional information which was not evident on CT scan. In patient #3, CT scan was normal but MRI revealed striatopallidodentate and mid brain T1 hyperintensity, highlighting greater sensitivity of MRI over CT scan. However, in patient #1 right frontal subcortical calcification was not evident on MRI. These results confirm the diversity of signal changes in PsHP; however, T1 hyperintensity in striatopallidodentate region is the most common pattern in PsHP.

Hyperintensity on T1 in calcification is attributed to surface interaction of protons with calcified tissue. At lower concentration of calcium, T1 shortening results in hyperintensity, whereas at high concentration (above 30-40%) susceptibility effects and decrease in proton density dominate leading to signal intensity loss. High signal intensity was typically a band around the periphery of affected area giving way to area of markedly reduced signal intensity. The intensity in center corresponds to the most heavily calcified tissue. In this area, reduction of proton density is the prime determinant of signal intensity. This hypothesis was supported by experimental studies.[4]

Our results confirm the importance of T1 hyperintensity in PsHP, and sequential long-term follow-up MRI studies are needed to confirm the postulation about signal alternations in PsHP.
